# A unique case of diagnosis of a heterotopic pregnancy at 26 weeks – case report and literature review

**DOI:** 10.1186/s12884-020-03465-y

**Published:** 2021-01-18

**Authors:** Anna Kajdy, Katarzyna Muzyka-Placzyńska, Dagmara Filipecka-Tyczka, Jan Modzelewski, Marek Stańczyk, Michał Rabijewski

**Affiliations:** 1 Department of Reproductive Health, Centre of Postgraduate Medical Education, 90 Żelazna St, 01-044 Warsaw, Poland; 2 Department of General, Oncologic and Trauma Surgery, Wolski Hospital, Kasprzaka 17, 01- 211 Warsaw, Poland

**Keywords:** Acute abdomen, Ectopic pregnancy, Heterotopic pregnancy, Second-trimester diagnosis, Case report

## Abstract

**Background:**

Heterotopic pregnancy (HP) is a rare condition when at least two pregnancies are present simultaneously at different implantation sites and only one located in the uterine cavity. The majority of cases are diagnosed in the first trimester.

**Case presentation:**

We present a unique case of HP diagnosed at 26 weeks of spontaneous pregnancy in a patient without any relevant risk factors. We performed an extensive review of HP cases from MEDLINE (PUBMED) published in English between 2005-2019 to prove this case's uniqueness.

A 24-year-old woman presented because of threatened preterm birth. Despite treatment, pain aggravated, without progression of labor. An emergency ultrasound exam revealed free fluid in the abdominal cavity. Suspicion of active bleeding prompted the medical team to perform an exploratory laparotomy. The surgery team found a ruptured heterotopic pregnancy. This was an unexpected cause of nontraumatic hemoperitoneum at such advanced gestational age. The postoperative period was uneventful, and the intrauterine pregnancy continued to term.

The final review included 86 out of 124 records. A total number of 509 cases were identified, but not all of them had complete data. The maximum reported gestational age at the time of diagnosis was 16 weeks of pregnancy, while our case became symptomatic and was diagnosed at 26 weeks of pregnancy.

**Conclusions:**

Regardless of pregnancy age, HP can be a cause of hemoperitoneum, and it should be included in the differential diagnosis of acute abdomen in the second trimester.

**Supplementary Information:**

The online version contains supplementary material available at 10.1186/s12884-020-03465-y.

## Background

Heterotopic pregnancy (HP) is a rare condition where at least two pregnancies are present simultaneously at different implantation sites and one of them located in the uterine cavity. Its prevalence varies from 1 to 30 000 in a natural cycle to around 1 in 100 in an assisted ones [1]. Like in ectopic pregnancy, abdominal pain, vaginal bleeding with positive pregnancy test are the common syndromes, but the diagnosis is more complicated. Even in the era of high-resolution ultrasound imaging and Doppler techniques, most of the time, the diagnosis is based on the presence of acute abdominal symptoms.

In the first trimester, unrecognized heterotopic pregnancy can be a cause of nontraumatic acute abdomen. We present a unique case of heterotopic pregnancy recognized at 26 weeks of gestation when the patient presented with hemoperitoneum after tubal rupture. To our knowledge, this is the first reported case of such late symptomatic heterotopic pregnancy in the medical literature.

## Case presentation

Medical records of the patient were reviewed and reevaluated to write this case report. Bioethics Commission of the Centre of Postgraduate Medical Education issued permission for retrospective analysis of medical records (Reference number 47/PB/2018). The patient gave written consent for publication of this case report and prepared a written account of her experience.

We have used CARE guidelines to ensure proper reporting of our case.

We also performed a literature review to find similar cases because the final diagnosis was unexpected for our team. The critical question for us was, could we have suspected the definitive diagnosis. The aim was to find cases of late second or third-trimester heterotopic pregnancies and analyze their outcome. MEDLINE (PUBMED) database was searched with the following phrases: “heterotopic pregnancy,“ “ectopic pregnancy,“ “assisted reproduction technology,“ the same as previously described by Barrenetxea [2]. We have searched all case reports, case series, and cohort studies with at least one case report of heterotopic pregnancy published between 01.01.2005–31.01.2019.

A 24-year-old primipara presented at St. Sophia’s Specialist Hospital at 26 weeks because of regular uterine contractions of the uterus and upper abdominal pain. The cervical length on admission was 23 mm. She was hospitalized because of threatening preterm birth two weeks earlier and was discharged after symptoms subsided. The patient had no important medical and gynecological history, except for an episode of first trimester bleeding that resolved spontaneously. The patient was followed prenatally in a private facility separate from the hospital. Her ultrasounds before 12 weeks of pregnancy were unavailable for reassessment. On the 12 weeks scan, the adnexa were not assessed.

After admission, betamethasone was administered for fetal lung maturity, and atosiban tocolysis was initiated. Because of the imminent risk of premature delivery, the patient received magnesium sulfate for fetal neuroprotection. Upper abdominal pain and difficulties in breathing became progressively worse despite the analgesic treatment. The patient became hypotensive tachycardic. Cervical dilatation did not progress during observation. On physical examination, she presented with rebound tenderness and involuntary guarding. Ultrasound showed free abdominal fluid. A surgeon was called for suspicion of hemoperitoneum with hypovolemic shock, and the patient was qualified for emergent exploratory laparotomy. The initial suspicion was internal bleeding from other internal organs or vessels outside of the reproductive tract.

**Fig. 1:**
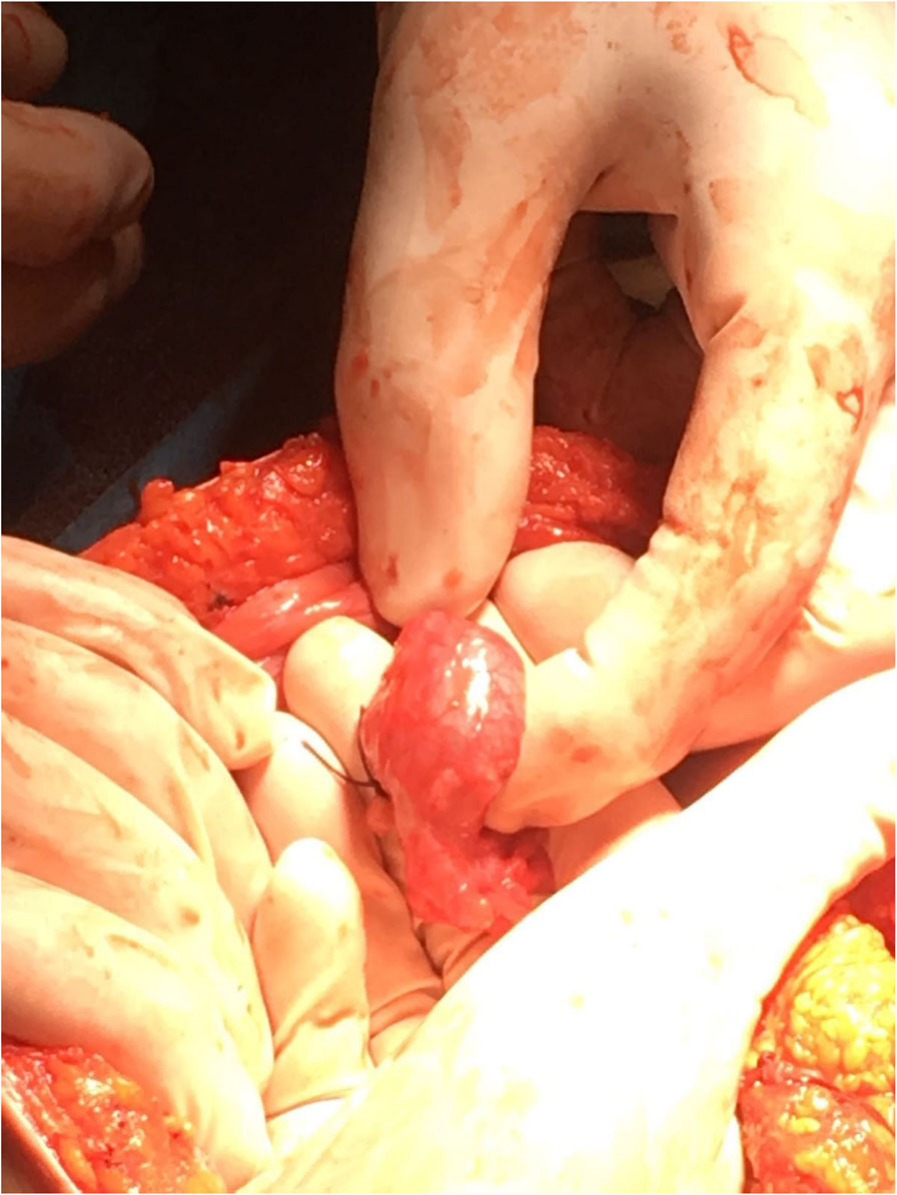
Enlarged fallopian tube

**Fig. 2:**
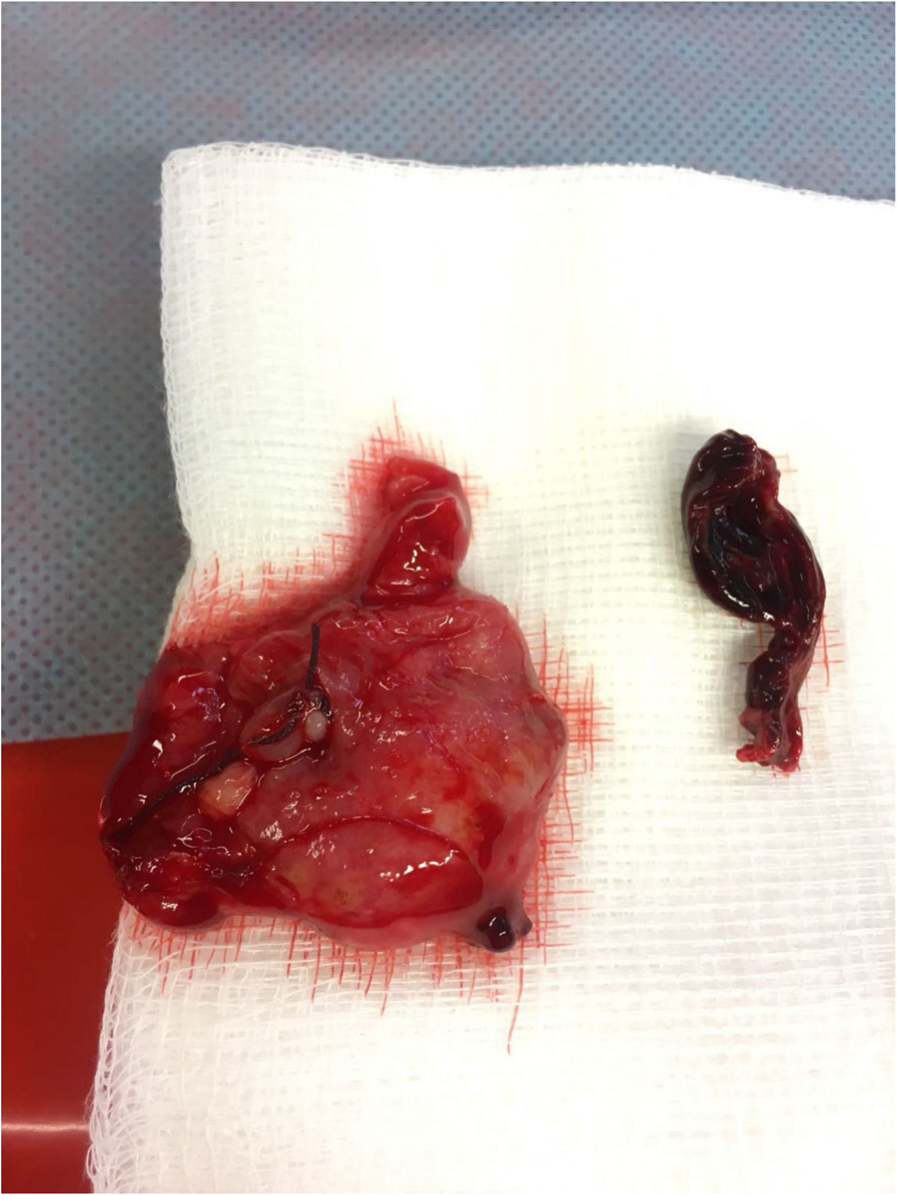
Resected fallopian tube with ectopic pregnancy

Following an inferior midline incision, 1000 ml of blood was removed by scooping and suction. In the left salpinx, there was a tumour compatible with a ruptured tubal pregnancy (Figs. 1 and 2). Left-sided salpingectomy and thorough inspection of all internal organs were performed. Intraoperative ultrasound confirmed fetal well-being. Petzer drain was placed in the abdominal cavity, and the patient received prophylactic antibiotics.

Four units of packed red blood cells and one fresh frozen plasma were transfused the same day without any complications. The histopathological exam confirmed ruptured salpinx with extrauterine pregnancy.

The wound healed without any complications. Follow up consultations were scheduled in the Perinatology Outpatient Clinic after discharge. The patient had psychological and psychiatric post-hospitalization counseling. Due to post-traumatic stress disorder, the patient was qualified for a planned, term cesarean section because of tokophobia. The rest of the pregnancy was uneventful. Although very anxious after treatment, the patient was grateful to the medical team for a justified intervention.

**Fig. 3:**
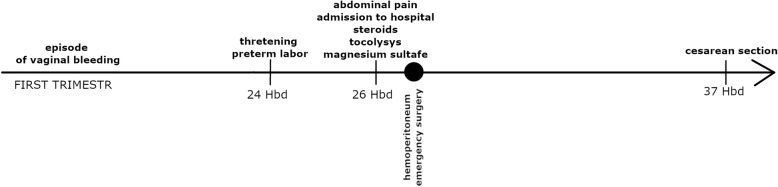
Timeline

She came to the hospital with regular contraction at 37 weeks of gestation. Cesarean section was performed, and a male neonate was delivered weighing 3580 grams. The procedure was uneventful; no intraperitoneal adhesions were found. The timeline of case events is presented in Fig. 3.

**Fig. 4:**
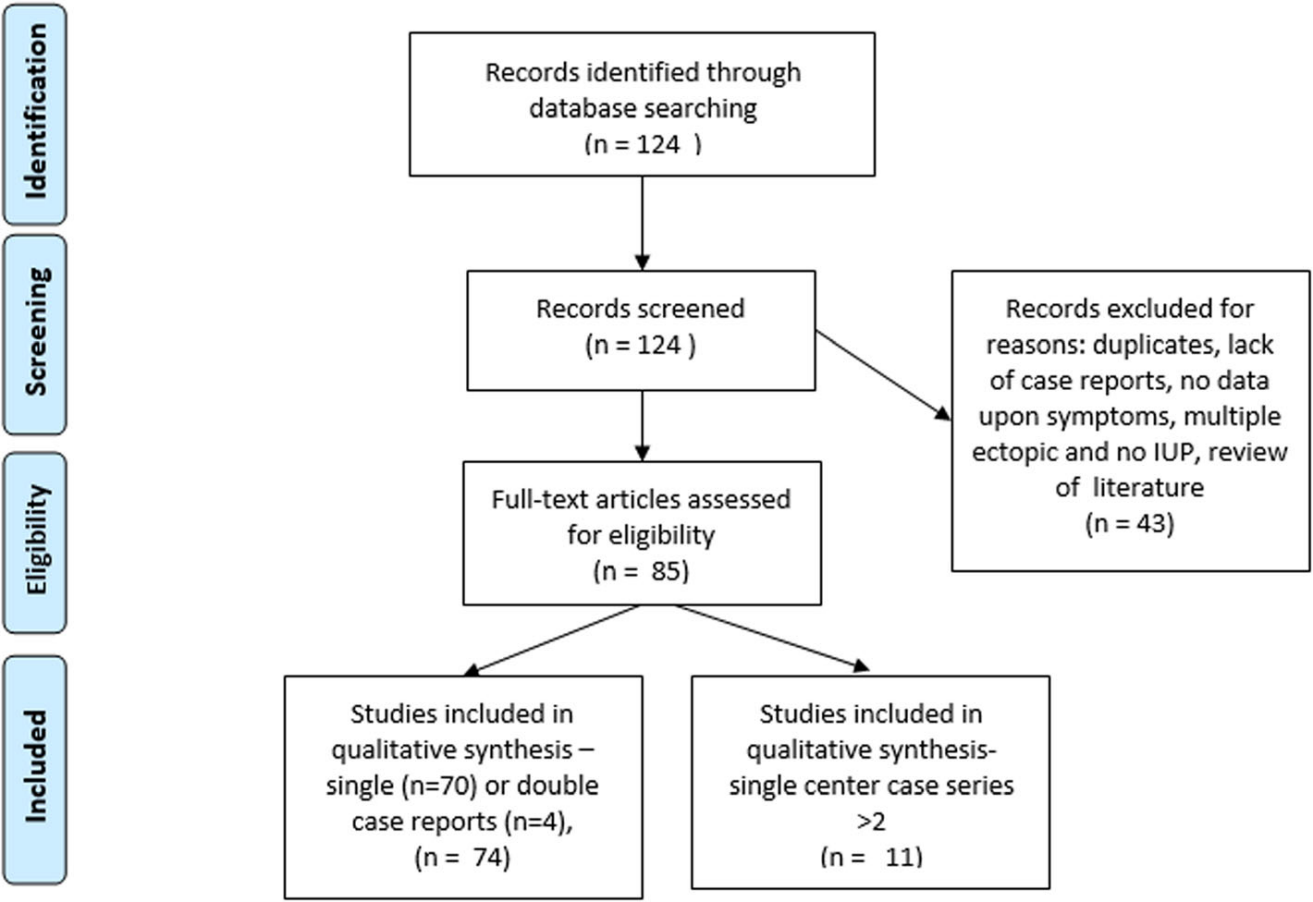
PRISMA flow chart of Medline Search

For the literature review, a total of 86 records were included in the final analysis. (Fig. 4) There were 75 single or double case reports, and 11 were single-center case series, with the largest including 132 confirmed heterotopic pregnancies [3].

Supplemental materials (Table 1) presents all papers and extracted data. The database includes gestational age at diagnosis, methods used for diagnosis, treatment modality, signs, symptoms, and pregnancy outcome. A total number of 509 cases were reviewed, but not all data were available for all cases.

**Fig. 5:**
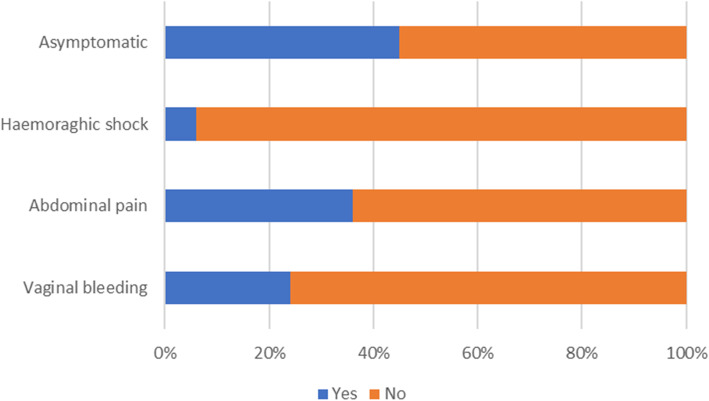
Frequency of symptoms in heterotopic pregnancy patients

The latest gestational age at the time of diagnosis was 16 weeks of pregnancy [4], and the median gestation age was seven weeks. Of those 509 cases, only 55% (280 cases) had described symptoms. Some of the patients had more than one symptom, but each symptom was counted separately. 45% (229 cases) of asymptomatic women were diagnosed by ultrasound examination. Among symptomatic patients, 66% had abdominal pain (185 cases), and 42% had vaginal bleeding (121 cases). The other described symptoms were less frequent, i.e., hemorrhagic shock, nausea, and vomiting were present in 28 and 8 women, respectively, 4 had a loss of consciousness. Figure 5 presents the frequency of symptoms in heterotopic pregnancy patients.

**Fig. 6:**
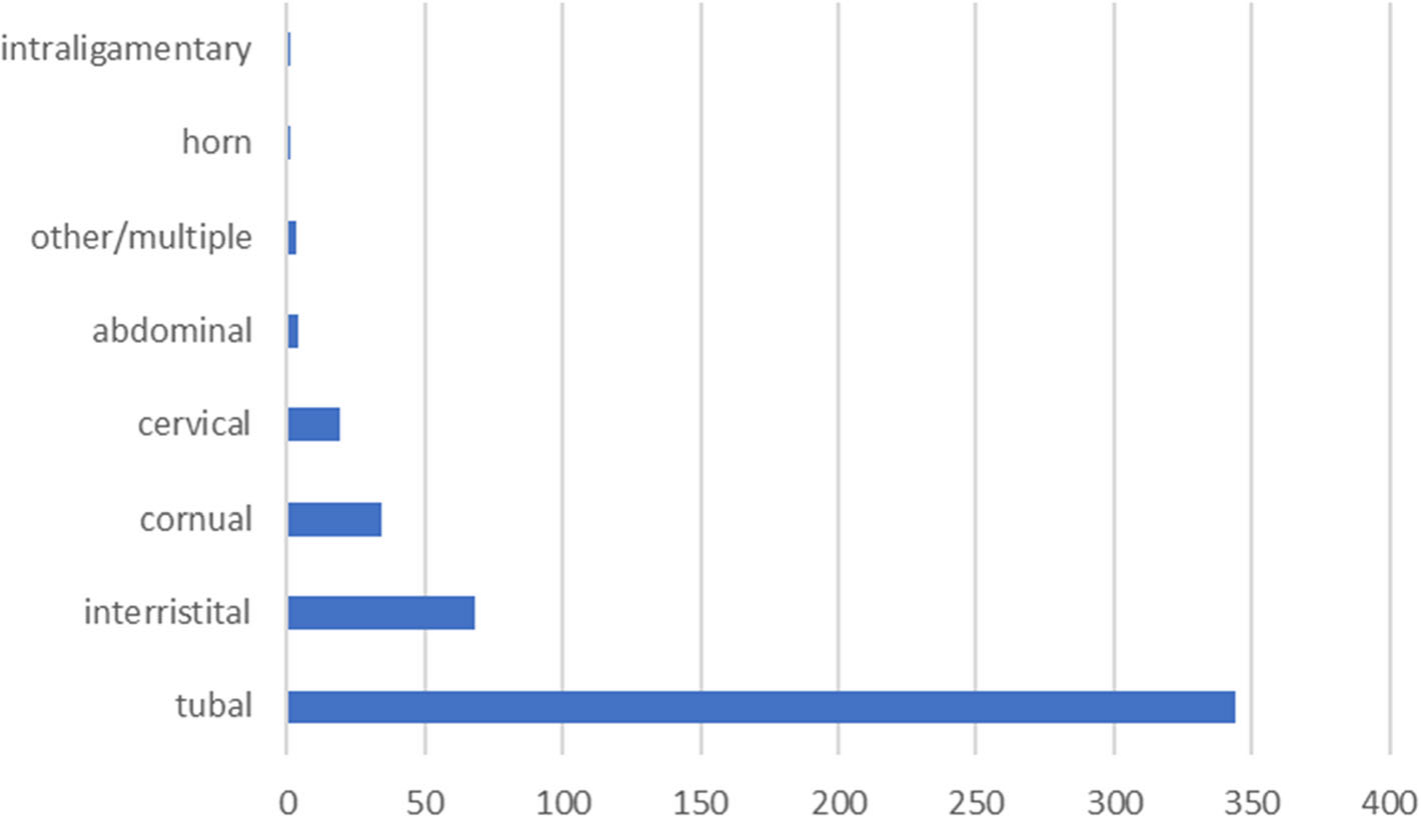
Location of heterotopic pregnancies

As shown in Fig. 6, tubal pregnancy was the most common location, while interstitial, cornual, and cervical ectopic pregnancies were less frequent.

**Fig. 7:**
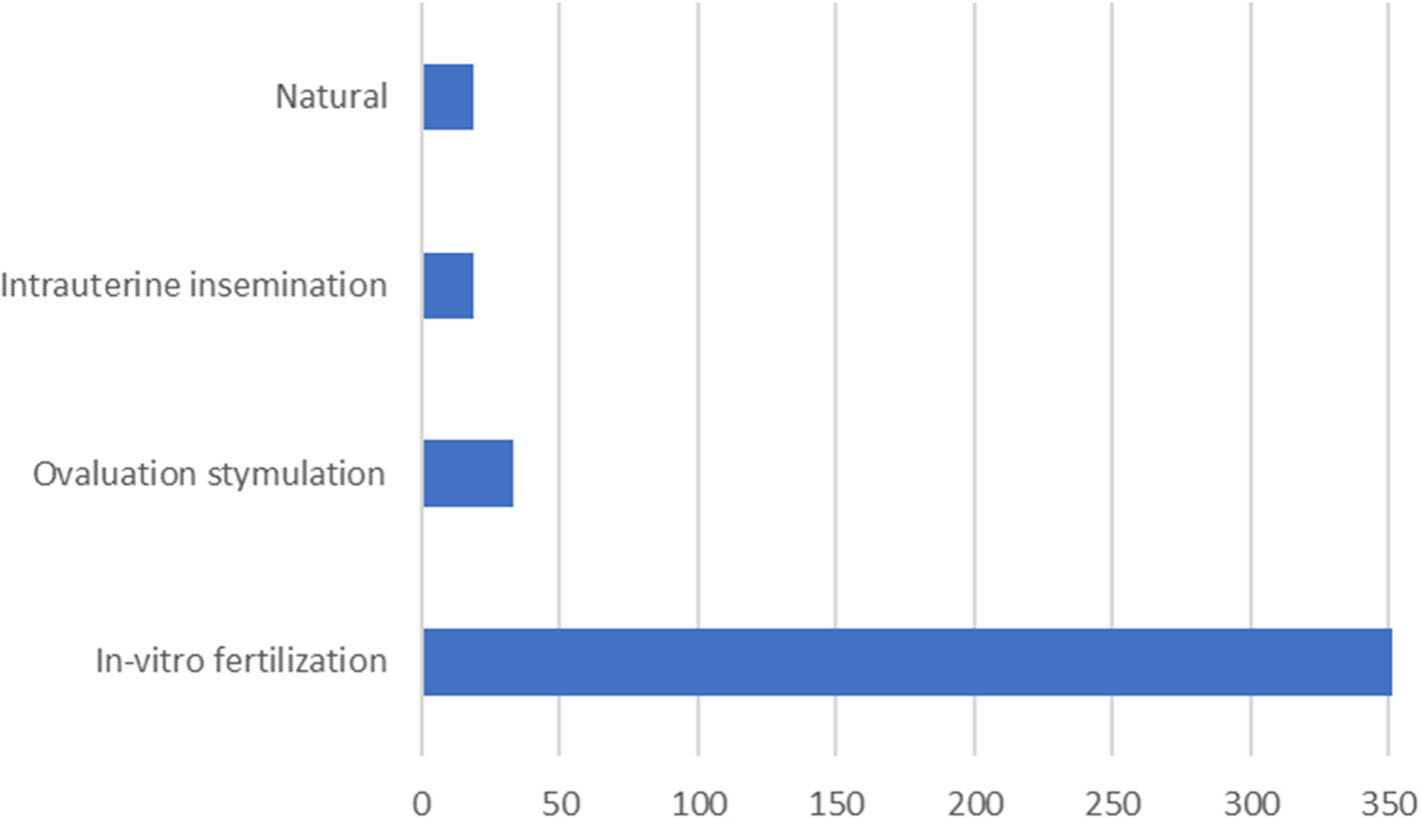
Method of conception of heterotopic pregnancies

In vitro fertilization (IVF) was the primary method of conception for women with heterotopic pregnancy (85%). Pregnancies after natural conception, ovarian stimulation, and intrauterine insemination occurred in 4%, 7%, and 4% of patients, respectively (Fig. 7). The outcome of intrauterine pregnancy was described in 85 cases. Intrauterine pregnancies ended with livebirth in 60% (51 cases). In 47 cases, we found information about pregnancy age at the time of labor. Preterm delivery occurred in 30% of them (14 women), while 70% delivered at term (33 women). Around 30% of pregnancies ended with miscarriage (26 cases), and 10% of patients decided to terminate intrauterine pregnancy. At the time of publication, 22 pregnancies of the screened records were ongoing.

## Discussion and conclusions

After spontaneous conception, the HP incidence is 1 per 30 000 pregnancies, but assisted reproductive techniques (ART) such as IVF and induction of ovulation contributed to higher rates. In the recent National ART Surveillance System bet ween 2001 and 2011, which reported 553,577 pregnancies, only 485 heterotopic pregnancies were identified – that is 1 per 1111 [5]. Our patient had a spontaneous conception.

HP’s risk factors are ART, especially IVF in fresh, non-donor cycles or multiple embryo transfer [5]. Also, a history of extrauterine pregnancy, previous surgery (including salpingectomy, salpingostomy, or reconstructive tubal surgery), and a history of pelvic inflammatory disease predispose to HP. In our case, the patient had neither of those risk factors, which makes the case even more unique.

In the review of the published literature from January 1994 to December 2004, performed by Barrenetxea, 13 HP cases were spontaneous, and nearly 74% were diagnosed early, between 5 and 8 weeks of gestation. However, there was one case recognized at 20 weeks [2]. Our data is similar: 14 spontaneous HPs and mostly diagnosed between 6 and 8 weeks, and our reported case was the oldest one (26 weeks).

Most HPs were diagnosed during surgery, either laparoscopy or laparotomy (59–74%) [2] the rest by ultrasonography. Barrenetxea’s report of HP term delivery occurred in 62,5%, preterm in 6%, and 31% of gestations ended with a miscarriage of intrauterine pregnancy [2]. In our review, 60% ended with livebirth, but 22 pregnancies were ongoing at publication.

Comparing the frequency of signs, the most frequent was pain (36%), the second was vaginal bleeding (24%), which mimics miscarriage symptoms and explains the difficulties with early diagnosis.

Our case report’s weak side is no ultrasound documentation of salpinx and ovaries from the first trimester. It would be interesting to assess the images retrospectively. Our obstetrics unit does not have magnetic resonance or computed tomography on site. Therefore, this was strictly a clinical diagnosis. Our literature review also suffers from incomplete data. The reviewed papers were missing descriptions of symptoms, complications, number of embryos transferred in a case of IVF, or intrauterine pregnancy results. Some were written before the delivery, making analysis of the risk of preterm delivery impossible. For this reason, reporting according to CARE guidelines is so important.

The study’s strong side is that it presents a well-managed clinical diagnosis of hemoperitoneum with an excellent outcome. In the differential diagnosis of hemoperitoneum, the main question is: was it caused by trauma. After excluding an injury, iatrogenic causes such as anticoagulation therapy, invasive procedures, or surgery in anamnesis should be considered. Spontaneous nontraumatic hemoperitoneum may result from hemorrhage from an ovarian cyst or vascular lesions such as an arterial aneurysm or even neoplasm rupture. In women of childbearing age, complications of early pregnancy should be taken into consideration. Ectopic pregnancy or much less common heterotopic pregnancy can cause dramatic internal hemorrhage, especially in a woman with preexisting risk factors.

The main “take-away” lesson from this case report is that adnexa should be routinely examined during the first-trimester scan. Our patient had first trimester bleeding, but HP was not recognized. The differential diagnosis of internal hemorrhage in the late second trimester should include ruptured heterotopic pregnancy, even in a potentially low-risk patient.

## Supplementary Information

**Additional file 1: Table 1.** List of case reports of heterotopic pregnancies used in the analysis.

## Data Availability

The data and materials are available on request from the corresponding author.

## Abbreviations

HP: Heterotopic pregnancy
IVF: In vitro fertilization
ART: Assisted reproductive techniques
